# Molecular mechanisms and therapeutic strategies: DNA methylation in pancreatic cancer

**DOI:** 10.1080/15384047.2026.2644667

**Published:** 2026-03-25

**Authors:** Junlin Liu, Junhua Xie, Yunyu Ding

**Affiliations:** a Department of Pathology, Jingmen Central Hospital, Hubei, People's Republic of China; b Department of Pathology, Jingmen Central Hospital Affiliated to Jingchu University of Technology, Hubei, People's Republic of China; c Department of Neurology, Jingmen Central Hospital, Hubei, People's Republic of China; d Department of Neurology, Jingmen Central Hospital Affiliated to Jingchu University of Technology, Hubei, People's Republic of China

**Keywords:** Pancreatic cancer, DNA methylation, epigenetics, biomarkers, epigenetic therapy

## Abstract

**Objective:**

This narrative review examines DNA methylation dysregulation in pancreatic cancer, focusing on its mechanistic roles in tumorigenesis and applications in biomarkers and therapies.

**Methods:**

We analyzed experimental and clinical studies on aberrant DNA methylation patterns, emphasizing their links to invasion, metastasis, therapy resistance, and immune modulation.

**Results:**

Pancreatic cancer exhibits recurrent tumor suppressor gene hypermethylation and locus-specific hypomethylation, driving progression and resistance. Methylation-based biomarkers (tissue/cfDNA) show promise for early detection and prognostication, though evidence remains largely retrospective or preclinical.

**Discussion:**

DNA methyltransferase inhibitors, alone or combined with immunotherapy/targeted agents, demonstrate preclinical and early clinical efficacy. Key challenges include mechanistic gaps, tumor heterogeneity, and assay standardization.

**Conclusion:**

DNA methylation is a actionable regulatory layer in pancreatic cancer, but further mechanistic and clinical validation is needed for translational impact.

## Graphical abstract

**Figure:**
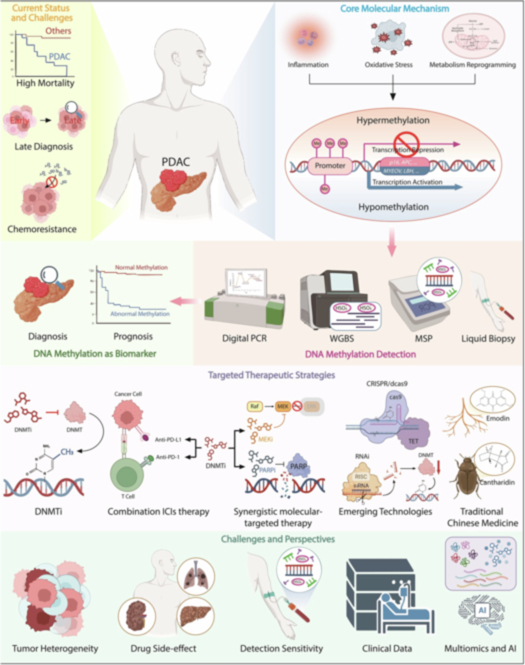

## 1. Introduction

Pancreatic cancer is a highly aggressive malignancy whose global incidence and mortality have continued to rise, placing it among the leading causes of cancer-related death worldwide. According to recent GLOBOCAN estimates, pancreatic cancer accounts for approximately 511,000 new cases and 467,000 deaths annually, with a five-year survival rate of around 10%; most patients die within one year of diagnosis.^1^ The exceptionally poor prognosis is largely attributable to nonspecific early clinical manifestations, which result in delayed diagnosis at advanced stages and limit opportunities for curative surgical intervention.^2^^,^^3^ Even among patients eligible for early resection, long-term survival remains unsatisfactory, in part due to the early development of micrometastatic disease.^2^^,^^4^ Conventional treatments, including chemotherapy and radiotherapy, provide only modest benefit, and although immunotherapy has generated considerable interest, durable survival improvements in pancreatic cancer remain limited.^2^^,^^5^ Consequently, there is a pressing need to identify novel molecular mechanisms that can support improved diagnostic, prognostic, and therapeutic strategies.

Epigenetic regulation, defined as heritable yet reversible changes in gene expression that occur without alterations in DNA sequence, plays a central role in cellular homeostasis and disease pathogenesis.^6^ Epigenetic mechanisms include DNA methylation, histone modifications, and non-coding RNA regulation, which collectively shape transcriptional programs governing cell differentiation, genomic stability, and stress responses.^7-11^ Among these mechanisms, DNA methylation has emerged as a key regulatory layer in cancer biology.^12^^,^^13^ Aberrant DNA methylation patterns in cancer are typically characterized by promoter hypermethylation of tumor suppressor genes, leading to transcriptional silencing, alongside genome-wide or locus-specific hypomethylation that contributes to genomic instability and oncogene activation.^12^^,^^14^^,^^15^ Increasing evidence suggests that these alterations are not merely epiphenomena but actively contribute to tumor initiation, progression, metastatic potential, and therapeutic resistance.^16^^,^^17^ From a clinical perspective, DNA methylation alterations hold potential across three major domains: early diagnosis to distinguish malignant disease from benign conditions such as chronic pancreatitis; prognostic stratification to identify high-risk patient subgroups; and predictive biomarker development to guide the selection of chemotherapy, targeted therapy, or immunotherapy.

The reversible nature of DNA methylation has also positioned epigenetic modulation as an appealing therapeutic strategy within precision oncology. DNA methyltransferase inhibitors (DNMTis) have demonstrated established clinical efficacy in hematologic malignancies and have shown emerging activity in preclinical models and early-phase studies of solid tumors, including pancreatic cancer.^12^^,^^14^^,^^18^ In parallel, advances in gene-editing technologies, such as CRISPR-based epigenetic editing, have enabled increasingly precise manipulation of DNA methylation states, further expanding the conceptual therapeutic landscape.^19^ Nevertheless, translation of methylation-targeted approaches into routine pancreatic cancer care remains constrained by tumor heterogeneity, incomplete mechanistic understanding, off-target effects, and the absence of robust clinical validation.^18^^,^^20^

In this narrative review, we synthesize representative mechanistic, translational, and emerging clinical evidence on DNA methylation dysregulation in pancreatic cancer. We focus on three interrelated aspects: (i) molecular mechanisms by which aberrant DNA methylation contributes to pancreatic cancer pathogenesis; (ii) the potential and limitations of methylation-based biomarkers across diagnostic, prognostic, and predictive settings; and (iii) current progress and challenges in therapeutic strategies targeting DNA methylation, including combination approaches. By integrating these perspectives, this review aims to provide a balanced framework for understanding both the opportunities and limitations of DNA methylation–directed research and to highlight priorities for future investigation in pancreatic cancer.

## 2. Molecular mechanisms of DNA methylation

### 2.1. Fundamental principles and regulatory enzymes of DNA methylation

DNA methylation is a critical epigenetic modification that involves the covalent addition of a methyl group to the fifth carbon of cytosine, generating 5-methylcytosine (5 mC), predominantly at CpG dinucleotide sites.^21-24^ This process is catalyzed by the DNA methyltransferase (DNMT) family of enzymes and represents a fundamental mechanism regulating gene expression, genomic stability, and cell identity.^25^ In mammalian cells, the DNMT family comprises three principal members—DNMT1, DNMT3A, and DNMT3B—with distinct but complementary biological functions.

**Figure 1.**
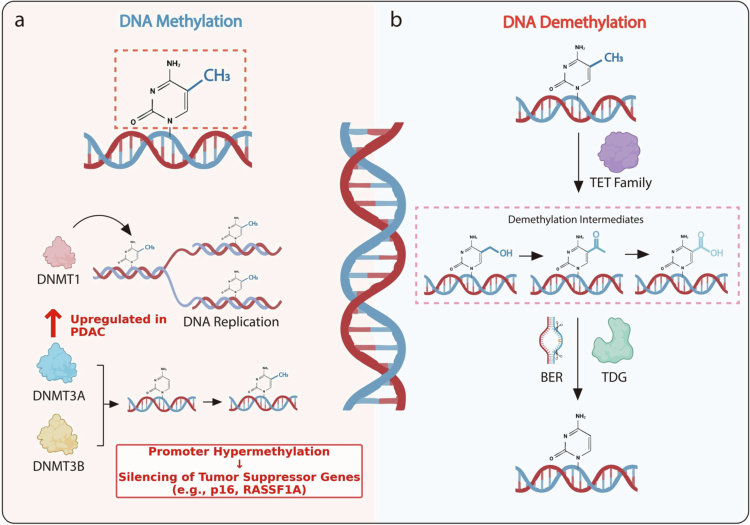
DNA Methylation ProcesDNA methylation and demethylation machinery and their dysregulation in pancreatic ductal adenocarcinoma (PDAC). (a) DNA methylation. DNA methylation is a key epigenetic modification involving the addition of a methyl group to the fifth carbon of cytosine (5-methylcytosine, 5 mC), predominantly at CpG dinucleotide sites. This process is catalyzed by DNA methyltransferases (DNMTs). DNMT1 primarily maintains methylation patterns during DNA replication and is frequently reported to be upregulated in PDAC, where it is associated with promoter hypermethylation and transcriptional silencing of tumor suppressor genes (e.g., p16, RASSF1A). In contrast, DNMT3A and DNMT3B are responsible for de novo methylation during development and cellular differentiation. (b) **DNA demethylation.** Active DNA demethylation is mainly mediated by the Ten–Eleven Translocation (TET) family of enzymes, which oxidize 5 mC to 5-hydroxymethylcytosine (5hmC), 5-formylcytosine (5fC), and 5-carboxylcytosine (5caC). These intermediates are subsequently excised by thymine DNA glycosylase (TDG) and repaired through the base excision repair (BER) pathway, ultimately restoring unmethylated cytosine.

DNMT1 primarily functions as a maintenance methyltransferase, ensuring faithful replication of pre-existing methylation patterns from parental DNA strands to newly synthesized daughter strands during DNA replication, thereby preserving epigenetic stability across cell divisions.^26^ In contrast, DNMT3A and DNMT3B act as de novo methyltransferases, establishing new methylation marks on previously unmethylated genomic regions. This activity is particularly important during embryonic development, lineage specification, and cell fate determination, when extensive epigenetic reprogramming occurs (Figure 1A).^27^^,^^28^

In addition to methylation, regulated DNA demethylation is essential for maintaining epigenetic plasticity. DNA demethylation is generally understood to occur through two mechanistically distinct pathways: passive and active demethylation.^29^ Passive demethylation refers to the gradual dilution of 5 mC during successive rounds of DNA replication when maintenance methylation by DNMT1 is impaired or absent.^30^ Active demethylation, by contrast, is mediated by the Ten–Eleven Translocation (TET) family of dioxygenases, which catalyze the stepwise oxidation of 5 mC to generate 5-hydroxymethylcytosine (5hmC), 5-formylcytosine (5fC), and 5-carboxylcytosine (5caC).^31-34^ These oxidized cytosine derivatives are subsequently recognized and excised by thymine DNA glycosylase (TDG) and replaced with unmodified cytosine through the base excision repair (BER) pathway, thereby completing the active demethylation process (Figure 1B).^30^^,^^35-40^

The activity, localization, and genomic targeting of DNMT and TET enzymes are tightly regulated by a range of intrinsic and extrinsic factors, including cellular metabolic state, availability of methyl donors, environmental exposures, and intracellular signaling pathways. Disruption of this finely balanced methylation–demethylation machinery can result in widespread epigenetic dysregulation. In pancreatic cancer, accumulating evidence indicates that altered DNMT and TET activity is frequently associated with aberrant gene silencing, epigenetic instability, and malignant progression, providing a mechanistic foundation for the methylation abnormalities discussed in subsequent sections.

### 2.2. Types of DNA methylation aberrations and their oncogenic mechanisms

DNA methylation aberrations in cancer predominantly manifest as two interrelated patterns: promoter hypermethylation and genome-wide hypomethylation.

Promoter hypermethylation frequently occurs at CpG islands within the promoters of tumor suppressor genes. In pancreatic cancer, this epigenetic alteration is commonly associated with transcriptional silencing of key regulatory genes, including p16, APC, RASSF1A, and E-cadherin. Such epigenetic repression has been linked to disruption of essential cellular processes, including cell cycle checkpoint control, apoptosis, and cell–cell adhesion, thereby contributing to a cellular environment permissive for tumor initiation and progression.^41^^,^^42^

In contrast, genome-wide hypomethylation is characterized by a global reduction in DNA methylation levels, particularly across intergenic regions and repetitive elements. This epigenetic state has been closely associated with genomic instability, including increased susceptibility to chromosomal rearrangements, insertions, and deletions, as well as elevated mutational burden that fosters intratumoral heterogeneity.^43^^,^^44^ Moreover, hypomethylation may lead to aberrant activation of proto-oncogenes or normally silenced genomic elements, such as endogenous retroviral elements (ERVs). Reactivation of ERVs has been proposed to generate double-stranded RNAs that mimic viral infection (“viral mimicry”), a process that may influence inflammatory signaling and immune modulation within the tumor microenvironment.^45^

### 2.3. Upstream signals influencing methylation status

Inflammation, oxidative stress, and metabolic reprogramming play pivotal roles in shaping DNA methylation landscapes in pancreatic cancer. Chronic inflammation, a key driver of tumor initiation—particularly in the context of pancreatitis—has been shown to influence the expression and activity of DNA methyltransferases (DNMTs). This regulation is often mediated by inflammatory cytokines and signaling pathways and is associated with aberrant promoter hypermethylation, leading to transcriptional silencing of tumor suppressor genes or dysregulation of oncogene expression.^46^

Oxidative stress further contributes to epigenetic instability in pancreatic cancer. Elevated levels of reactive oxygen species (ROS) can disrupt epigenetic homeostasis by inducing DNA damage that recruits DNMTs to repair sites or by impairing the activity of TET family demethylases. These ROS-associated alterations in DNA methylation are thought to exacerbate transcriptional dysregulation and support tumor progression.^47^

Metabolic reprogramming provides an additional mechanistic link between cellular metabolism and epigenetic regulation in pancreatic cancer. Under hypoxic conditions and increased metabolic demand, tumor cells alter glucose and amino acid metabolism, leading to the accumulation of oncometabolites such as L-2-hydroxyglutarate (L-2HG). L-2HG competitively inhibits *α*-ketoglutarate–dependent dioxygenases, including TET DNA demethylases, thereby promoting global DNA hypermethylation and contributing to the maintenance of stem-like phenotypes and immune evasion.^48^^,^^49^ Moreover, the expression of metabolic enzymes such as PHGDH is dynamically regulated by promoter methylation, highlighting a bidirectional interaction between metabolism and epigenetic control. Metabolic stressors, including nutrient deprivation, may further induce adaptive demethylation events that enhance tumor cell survival in resource-limited microenvironments.^49^

## 3. Detection methods for DNA methylation and their applications

A variety of techniques are available for detecting DNA methylation, each offering distinct advantages and limitations for research and clinical applications. Commonly used methods include whole-genome bisulfite sequencing (WGBS), methylation-specific PCR (MSP), and digital PCR–based assays. WGBS provides single–base-pair resolution methylation profiles and is therefore well suited for discovery-phase studies aimed at comprehensively mapping methylation landscapes in pancreatic cancer.^50^ However, its clinical utility is constrained by high costs, substantial computational demands, and the requirement for high-quality DNA input, which can be challenging when using formalin-fixed paraffin-embedded (FFPE) samples. MSP is a rapid and highly sensitive method frequently employed to assess the methylation status of predefined loci in both tissue and liquid biopsy samples,^51^ although it is inherently qualitative and limited to targeted regions. Digital PCR, by contrast, enables absolute quantification with high analytical sensitivity and is particularly advantageous for low-abundance circulating cell-free DNA (cfDNA). In this context, multiplexed digital PCR–based approaches have demonstrated promising potential, with some studies reporting detection of up to 120 methylation markers in cfDNA for early pancreatic cancer detection.^52^

Recent advances in high-throughput technologies, including next-generation sequencing (NGS)–based methylation profiling and array-based platforms, have substantially expanded the characterization of aberrant methylation patterns in pancreatic cancer. Large-scale methylome studies have systematically identified differentially methylated regions and genes associated with tumor initiation and progression, thereby establishing a foundation for biomarker discovery and mechanistic investigation.^53^^,^^54^ In parallel, third-generation sequencing technologies have enabled the exploration of emerging DNA modifications beyond canonical 5-methylcytosine, such as N6-methyladenine (6mA). Although several studies have reported elevated 6mA levels in pancreatic cancer and correlations with oncogenic pathways, the biological significance and clinical relevance of this modification remain under active investigation, and further mechanistic and translational validation is required.^55^

Liquid biopsy–based analysis of cfDNA methylation has attracted particular interest because of its minimally invasive nature and suitability for longitudinal monitoring. Diagnostic models based on cfDNA methylation signatures have demonstrated encouraging performance in distinguishing pancreatic cancer patients from healthy individuals, including improved sensitivity for early-stage disease compared with mutation-based assays.^56^ Integration of cfDNA methylation markers with established serum biomarkers, such as CA19-9, has further enhanced diagnostic accuracy in some cohorts. More recently, artificial intelligence (AI) and deep learning algorithms have been applied to cfDNA methylation datasets to construct diagnostic classifiers. While pilot studies have reported exceptionally high sensitivity and specificity within specific datasets, these performance metrics are often derived from relatively small, controlled cohorts. Independent external validation in larger and more heterogeneous populations, including appropriate benign disease controls such as chronic pancreatitis, is essential to mitigate overfitting and ensure clinical specificity before routine implementation.^57^

Despite these advances, several technical and translational challenges remain. Concordance between tissue-based and plasma-based methylation profiles has been reported for selected key markers but is influenced by tumor burden and variable cfDNA shedding rates. In addition, pre-analytical factors—including blood collection tubes, processing time before plasma separation, and leukocyte DNA contamination—can substantially affect assay performance and background noise. Consequently, the establishment of standardized operating procedures for sample collection, processing, and data analysis represents a critical prerequisite for the widespread clinical adoption of DNA methylation–based assays in pancreatic cancer.^58^^,^^59^

## 4. The role of DNA methylation in pancreatic cancer

### 4.1. Promoter methylation of tumor suppressor genes

**Figure 2.**
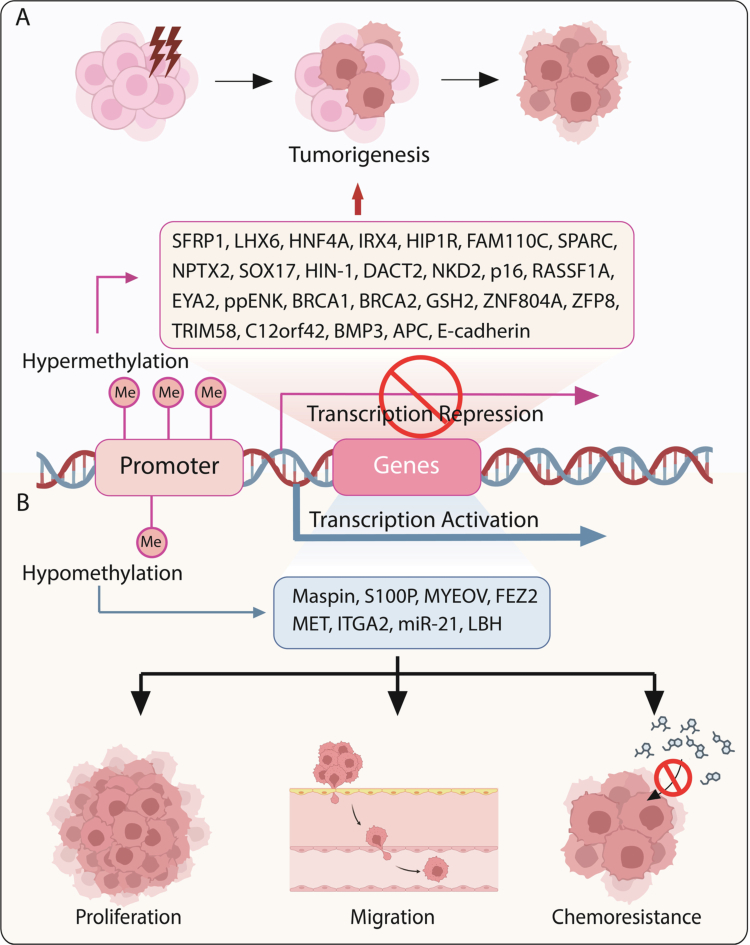
Aberrant DNA methylation of key genes in pancreatic cancer pathogenesis. (A) Promoter hypermethylation. Promoter hypermethylation represents a major epigenetic mechanism leading to the transcriptional silencing of tumor suppressor genes in pancreatic cancer. Based on the current body of evidence, frequently reported targets include genes with consistent validation across multiple clinical cohorts (e.g., p16, APC, E-cadherin, RASSF1A, BRCA1, and BRCA2), as well as additional candidates identified in preclinical studies or single-center clinical analyzes (e.g., SFRP1, LHX6, HNF4A, IRX4, HIP1R, FAM110C, SPARC, NPTX2, SOX17, HIN1, DACT2, NKD2, EYA2, ppENK, GSH2, ZNF804A, ZFP82, TRIM58, C12orf42, and BMP3). The epigenetic silencing of these genes is associated with tumor initiation and malignant progression. (B) Promoter hypomethylation. In contrast, promoter hypomethylation affects a distinct set of oncogenic drivers, including protein-coding genes (Maspin, S100P, MYEOV, FEZ2, MET, and ITGA2) as well as oncogenic microRNAs (e.g., miR-21). Hypomethylation-mediated transcriptional activation of these targets is primarily associated with aggressive phenotypes such as enhanced proliferation, migration, and chemotherapy resistance in pancreatic cancer.

Aberrant promoter methylation of tumor suppressor genes plays a critical role in the initiation and progression of pancreatic cancer (Table 1, Figure 2). Importantly, it is necessary to distinguish methylation events that function as drivers of tumorigenesis from those that represent secondary or passenger associations. During pancreatic carcinogenesis, promoter hypermethylation of key tumor suppressor genes constitutes a frequent and biologically meaningful epigenetic alteration. Genes such as p16 (CDKN2A), APC, and E-cadherin (CDH1) consistently exhibit high-frequency promoter methylation in pancreatic cancer. Notably, functional validation studies have demonstrated that pharmacological or genetic demethylation can restore the expression of these genes, supporting a causal role of epigenetic silencing in malignant progression.^28^^,^^42^^,^^60^^,^^61^

**Table 1.** DNA methylation markers associated with pathogenesis, metastasis, and prognosis of pancreatic cancer.

Gene(s)	Methylation Status	Specimen type	Assay method	Main clinical/biological association	Evidence level	References
*SFRP1, LHX6, HNF4A*	Hypermethylation	Tissue	MSP/qMSP	Aggressive tumor progression and poor survival	Clinical cohort	[62-64]
*IRX4, HIP1R, FAM110C*	Hypermethylation	Tissue/Cell lines	MSP/WB	Enhances tumor progression via signaling modulation	Preclinical	[65,66]
*SPARC, NPTX2*	Hypermethylation	Tissue	qMSP	Chemoresistance and adverse survival outcomes	Clinical cohort	[67]
*SOX17, HIN-1, DACT2, NKD2*	Hypermethylation	Tissue	MSP	Activation of oncogenic signaling and cell cycle progression	Clinical cohort	[68]
*p16(CDKN2A)*	Hypermethylation	Tissue/cfDNA	MSP/NGS	Cell cycle dysregulation; diagnostic and prognostic relevance	Large clinical cohorts	[61]
*RASSF1A*	Hypermethylation	Tissue/cfDNA	MSP	Chemoresistance and poor prognosis	Clinical cohort	[61]
*NPTX2, EYA2, ppENK*	Hypermethylation	Tissue/cfDNA	qMSP	Promotes pancreatic cancer progression	Clinical cohort	[69]
*BRCA1, BRCA2*	Hypermethylation	Tissue	MSP/NGS	Poor prognosis; BRCAness-associated phenotype	Clinical cohort	[70]
*GSH2*	Hypermethylation	Tissue	qMSP	Lymph node metastasis	Clinical cohort	[71]
*ZNF804A, ZFP8, TRIM58, C12orf42*	Hypermethylation	Tissue	Array/MSP	Independent predictors of poor survival	Clinical cohort	[72]
*BMP3*	Hypermethylation	Tissue/Stool	qMSP	Enhanced migration and invasion	Preclinical & clinical pilot	[73]
*APC*	Hypermethylation	Tissue	MSP	Wnt/β-catenin activation; proliferation and metastasis	Clinical cohort	[42]
*E-cadherin*	Hypermethylation	Tissue	MSP	EMT, migration, and invasion	Preclinical & clinical	[28]
*Maspin, S100P*	Hypomethylation	Tissue	MSP/IHC	Aberrant activation promotes tumor progression	Clinical cohort	[74]
*MYEOV*	Hypomethylation	Tissue/Cell lines	Bisulfite sequencing	Proliferation, metastasis, poor prognosis	Preclinical	[75]
*FEZ2*	Hypomethylation	Tissue	Array/PCR	Chemoresistance and migration	Preclinical	[76]
*MET, ITGA2*	Hypomethylation	Tissue	450 K array	Poor prognosis	Clinical cohort	[77]
*miR-21*	Hypomethylation	Tissue	MSP	Proliferation and invasion	Preclinical	[78]
*LBH*	Hypomethylation	Tissue	Array/RNA-seq	WNT–integrin signaling activation; poor prognosis	Clinical cohort	[79]

The cell cycle regulator p16 serves as a prominent example and is arguably the most consistently validated methylation marker in pancreatic cancer. Promoter hypermethylation of p16 has been observed in both tumor tissue and circulating tumor DNA in multiple independent studies and is closely associated with enhanced proliferative, migratory, and invasive capacities.^61^ Mechanistically, DNMT1 is frequently upregulated in pancreatic cancer and mediates coordinated methylation of several critical cell cycle regulators, including p16, p14, p15, p21, and p27, resulting in transcriptional repression and contributing to dysregulated differentiation and maintenance of stem-like properties.^28^

Beyond cell cycle control, promoter hypermethylation also disrupts key signaling pathways involved in tumor progression. The classical tumor suppressor APC frequently undergoes methylation-mediated silencing in pancreatic cancer, leading to aberrant activation of the Wnt/β-catenin pathway and promoting tumor cell proliferation and metastasis.^42^ Similarly, E-cadherin, a central regulator of cell–cell adhesion and an inhibitor of epithelial–mesenchymal transition (EMT), is epigenetically silenced via promoter methylation, thereby facilitating EMT, enhancing tumor cell motility, and fostering a more aggressive phenotype.^28^

Collectively, these findings highlight promoter hypermethylation of tumor suppressor genes as a core epigenetic mechanism driving pancreatic cancer progression and clinical heterogeneity. Additional methylation alterations summarized in Table 1—including both hypermethylation of tumor suppressors and hypomethylation-associated activation of oncogenic pathways—are included to provide a comprehensive overview of methylation dynamics relevant to pathogenesis, metastasis, and prognosis and are discussed in subsequent sections.

### 4.2. Hypomethylation and oncogene activation

DNA hypomethylation serves as an important epigenetic mechanism driving oncogene activation in pancreatic cancer. In contrast to global hypomethylation, which primarily contributes to genomic instability, promoter-specific hypomethylation affects genes that are normally epigenetically silenced in nonmalignant pancreatic tissue, leading to their aberrant transcriptional activation in tumor cells.^43-45^

Representative examples include Maspin and S100P, both of which exhibit promoter hypomethylation and increased expression in pancreatic cancer compared with normal pancreatic ducts.^74^ These features have prompted their investigation as potential diagnostic and disease-associated biomarkers rather than definitive clinical targets. Functional studies have further clarified the consequences of promoter demethylation–mediated oncogene activation. For instance, MYEOV (myeloma overexpressed) is activated in pancreatic ductal adenocarcinoma (PDAC) through promoter demethylation. This epigenetic derepression is associated with enhanced proliferative and metastatic capacity and has been linked to amplification of SOX9-dependent transcriptional programs, including upregulation of the downstream effector HES1, thereby contributing to malignant progression.^75^^,^^80^

Hypomethylation-driven oncogene activation is also closely linked to dysregulation of the Wnt signaling pathway. FEZ2 (Fasciculation and Elongation Protein Zeta 2), a methylation-regulated proto-oncogene, exhibits hypomethylation-associated overexpression in PDAC tissues and promotes tumor cell proliferation, migration, and chemotherapy resistance, at least in part through activation of Wnt signaling.^76^ Similarly, the developmental transcriptional cofactor LBH (Limb-Bud and Heart) is upregulated via promoter hypomethylation and contributes to activation of the WNT–integrin signaling axis. Increased LBH expression has been reported to correlate with adverse clinical outcomes in pancreatic cancer.^79^ In addition, large-scale methylation analyzes have identified hypomethylation of MET and ITGA2 as being associated with elevated gene expression and reduced patient survival, underscoring their potential prognostic relevance.^77^

Hypomethylation-mediated oncogene activation extends beyond protein-coding genes to include non-coding regulatory elements such as microRNAs. miR-21, a well-characterized oncogenic microRNA, is frequently overexpressed in pancreatic cancer as a consequence of promoter hypomethylation.^78^ Elevated miR-21 expression suppresses negative regulators of multiple signaling pathways, thereby enhancing tumor cell proliferation, invasion, and survival.

## 5. Targeted DNA methylation drugs and their therapeutic advances

### 5.1. DNA methyltransferase inhibitors

DNA methyltransferase inhibitors (DNMTis) are a class of epigenetic agents designed to reverse aberrant DNA methylation and restore the expression of tumor suppressor genes silenced by promoter hypermethylation (Table 2). Representative compounds, including 5-aza-2′-deoxycytidine (decitabine) and 5-azacytidine, function as cytidine analogs that become incorporated into DNA during replication. Upon incorporation, these agents form irreversible covalent complexes with DNMT enzymes, leading to enzyme depletion and sustained inhibition of both de novo and maintenance methylation. Through this mechanism, DNMTis can relieve transcriptional repression of hypermethylated tumor suppressor genes and suppress malignant cell proliferation.^14^^,^^81^^,^^82^

**Table 2.** Drugs targeting DNA methylation for pancreatic cancer treatment.

Agent	Experimental model	Effective dose	Key epigenetic mechanism and antitumor effects	References
5-Azacytidine	PANC-1, SW1990 cells	2.5 μM	Inhibits DNMT1 activity, leading to demethylation and reactivation of HIP1R, thereby suppressing proliferation, migration, and invasion while inducing apoptosis	[65]
Decitabine	Primary PDAC cells derived from surgically resected tumors	1 µM	Suppresses tumor growth by DNMT1 inhibition, reduces SOCS1 promoter methylation, restores SOCS1 expression, and blocks STAT3/IGF-1 signaling	[83]
Metformin + gemcitabine	BXPC-3 and MIAPaCa-2 cells	6.8 mM + 143.2 nM	Enhances gemcitabine sensitivity and reverses EMT through demethylation-mediated upregulation of miR-663	[84]
SGI-1027	Capan-2, MIAPaCa-2, HPAC, BXPC-3 cells	5 μM	Reverses CLDN1 promoter hypermethylation, restores gene expression, and suppresses tumor growth and invasiveness	[85]
zebularine	PA-TU-8902 cells; PaCa-44 cells	98.82 μM; 61.67 μM	Reactivates silenced tumor suppressor genes via DNA demethylation, inducing apoptosis through intrinsic and extrinsic pathways	[86]
CM272	MIA PaCa-2, PANC-1 cells	320 nM, 870 nM	Dual DNMT/G9a inhibition suppresses proliferation, induces apoptosis, and enhances chemosensitivity; Remodels tumor immune microenvironment and potentiates immune checkpoint blockade by increasing CD4⁺/CD8⁺ T-cell infiltration	[87]
	male C57/BL6 mice	5 mg/kg		

In pancreatic ductal adenocarcinoma (PDAC), accumulating preclinical evidence supports a mechanistic role for DNMTis in reversing gene-specific epigenetic silencing. The non-nucleoside DNMT inhibitor SGI-1027 has been shown in cell-based and xenograft models to reverse promoter hypermethylation of CLDN1, restore its expression, and suppress tumor growth and invasiveness.^85^ Similarly, *in vitro* studies demonstrate that decitabine and 5-azacytidine can demethylate and reactivate the organic cation transporter OCT2, thereby enhancing PDAC cell sensitivity to oxaliplatin.^88^ Notably, adaptive resistance to MEK inhibitors in pancreatic cancer has been linked to widespread DNA hypermethylation, and DNMTi treatment has been shown to partially reverse this resistance phenotype in preclinical models, highlighting the potential of epigenetic reprogramming to overcome targeted therapy resistance.^89^

Beyond direct effects on tumor cell–intrinsic pathways, DNMTis can remodel the tumor transcriptome and modulate the tumor microenvironment, potentially enhancing responsiveness to chemotherapy or immunotherapy.^90^^,^^91^ However, despite these promising biological effects, the clinical translation of DNMTis in solid tumors—including PDAC—has remained challenging. DNMTi monotherapy has historically demonstrated limited efficacy, largely due to dose-limiting toxicities, tumor heterogeneity, and the redundancy of epigenetic regulatory networks that enable rapid adaptive resistance.^92^

As a result, current therapeutic strategies increasingly emphasize rational combination approaches, including pairing DNMTis with histone deacetylase inhibitors (HDACis), targeted therapies, or immune checkpoint inhibitors, rather than monotherapy.^92-94^ Nevertheless, careful clinical implementation is required, as global DNA demethylation may, in certain biological contexts, inadvertently activate prometastatic or oncogenic programs.^95^ These considerations underscore the necessity for biomarker-guided patient selection and optimized dosing strategies to maximize therapeutic benefit while minimizing unintended epigenetic consequences.

### 5.2. Research progress of DNMTis in pancreatic cancer cell and animal models

In preclinical settings, DNMT inhibitors (DNMTis) have demonstrated significant antitumor activity in pancreatic cancer models. *In vitro* studies using multiple pancreatic cancer cell lines show that DNMTis, such as zebularine and decitabine, effectively suppress cell proliferation and induce apoptosis.^14^^,^^86^ Mechanistic analyzes further indicate that zebularine modulates the expression of apoptosis-related genes, partially activating both intrinsic and extrinsic apoptotic pathways. Notably, the dominant apoptotic dependencies appear to be cell line–specific, reflecting the epigenetic heterogeneity of pancreatic cancer.^96^

In animal models, emerging dual-targeting epigenetic strategies have shown enhanced efficacy. The dual inhibitor CM272, which simultaneously targets DNMT1 and the histone methyltransferase G9a, has demonstrated promising antitumor effects in mouse xenograft and syngeneic models. CM272 significantly inhibits tumor proliferation and induces apoptosis without overt systemic toxicity.^87^ Importantly, CM272 also remodels the tumor immune microenvironment, thereby increasing sensitivity to immunotherapy. When combined with immune checkpoint inhibitors, such as anti-PD-1 antibodies, CM272 augments antitumor immune responses by promoting CD4⁺ and CD8⁺ T-cell infiltration and activation. These findings highlight the potential of DNMT-targeting strategies as epigenetic immunomodulators and provide a strong rationale for further translational investigation.^87^

### 5.3. Traditional chinese medicine and DNA methylation

As a cornerstone of traditional medicine, Traditional Chinese Medicine (TCM) exhibits multi-target and multi-pathway pharmacological activities.^97^ Unlike synthetic single-target DNMT inhibitors, TCM interventions are proposed to modulate complex signaling networks, potentially influencing epigenetic regulation in an indirect or context-dependent manner. A growing body of preclinical research suggests that specific TCM formulations and purified bioactive components can regulate DNA methylation or related epigenetic processes (Table 3), offering exploratory therapeutic avenues for pancreatic cancer.

**Table 3.** Traditional Chinese Medicines Targeting DNA Methylation in Pancreatic Cancer Treatment.

Medicine	Model system & dosage	Evidence level	Key mechanisms	References
Emodin	Cells: PANC-1 (40 μM) Mice: BALB/c nude (20–80 mg/kg)	*In vitro*/*In vivo*	Directly downregulates DNMT1 and DNMT3A, leading to demethylation and reactivation of tumor suppressor genes (p16, RASSF1A, ppENK). Synergizes with 5-Aza-CdR; additionally suppresses angiogenesis via TGF-β/Smad signaling and related miRNAs.	[98-100]
QYHJ formula	Mice: BALB/c nude (36 g/kg)	*In vivo*	Indirect epigenetic regulation via suppression of Notch-4 and Jagged-1, modulating Notch signaling and inhibiting tumor growth.	[101]
Cantharidin	Cells: PANC-1, CFPAC-1 (20 μM)	*In vitro*	Suppresses invasion by promoting MMP2 mRNA degradation; epigenetic involvement suggested but not directly linked to DNA methylation.	[102]
Matrine	Cells: PANC-1, BxPC-3 (1.25–2 mg/mL); Mice: BALB/c nude (200 mg/kg)	*In vitro*/*In vivo*	Downregulates PCNA, activates intrinsic and extrinsic apoptotic pathways; inhibits migration via ROS/NF-κB/MMP axis, with indirect epigenetic modulation.	[103,104]
Linderalactone	Cells: ASPC-1, BXPC-3, CFPAC-1, SW-1990 (60 μM); Mice: BALB/c nude (50 mg/kg)	*In vitro*/*In vivo*	Induces G2/M cell cycle arrest and apoptosis; epigenetic regulation likely secondary to cell-cycle control.	[105]
Scoparone	Cells: CAPAN-2, SW-1990 (200 μM); Mice: BALB/c nude (200 μM, 50 μL)	*In vitro*/*In vivo*	Inhibits PI3K/AKT signaling, induces cell cycle arrest and apoptosis; indirect effects on epigenetic landscape.	[106]
Tanshinones	Cells: PANC-1, BXPC-3 (10 μM); Mice: C57BL/6 (30 mg/kg)	*In vitro*/*In vivo*	Activates AKT/FOXO3/SOD2 pathway, elevates ROS, induces apoptosis; epigenetic regulation likely ROS-mediated.	[107]
Huaier	Cells: MiaPaCa-2, PANC-1 (90 μg/mL); Mice: BALB/c nude (50–100 mg/kg)	*In vitro*/*In vivo*	Downregulates FoxM1 expression and nuclear translocation, reverses gemcitabine-induced stemness; indirect epigenetic reprogramming.	[108]
Betulinic acid	Cells: BXPC-3 (20 μM)	*In vitro*	Synergizes with gemcitabine; induces DNA damage and Chk1 proteasomal degradation; epigenetic effect not primary.	[109]
Bufalin	Cells: BXPC-3, SW-1990, PANC-1 (80 nM); Mice: BALB/c nude (2 mg/kg)	*In vitro*/*In vivo*	Downregulates c-Myc and inhibits HIF-1α/SDF-1/CXCR4 axis; indirect epigenetic influence via transcriptional control.	[110]
Huachansu	Mice: BALB/c nude (40 g/kg)	*In vivo*	Modulates TGF-β/Smad pathway, remodels tumor microenvironment; epigenetic effects inferred but not directly validated.	[111]
Isobavachalcone	Cells: PANC-02 (20 μM); Mice: C57BL/6 (20 mg/kg)	*In vitro*/*In vivo*	Induces apoptosis and enhances anti-tumor immunity by reducing M2 macrophages and MDSCs; immune-epigenetic interaction.	[112]
Panax notoginseng Saponins	Cells: Miapaca-2, PANC-1 (377–492 μM)	*In vitro*	Enhances gemcitabine sensitivity by promoting apoptosis and suppressing autophagy; synergistic, indirect epigenetic modulation.	[113]

Mechanistically, available evidence indicates that these agents may function through two principal modes. First, direct epigenetic modulation has been demonstrated for a limited number of compounds. For example, emodin directly downregulates DNMT expression and activity, leading to promoter demethylation and reactivation of tumor suppressor genes such as p16, RASSF1A, and ppENK. Second, a broader group of TCM agents exerts indirect epigenetic regulation by modulating upstream signaling pathways—including TGF-*β*, NF-κB, and PI3K/AKT—which are known to interact with chromatin modifiers and shape the epigenetic landscape. Together, these findings enrich the mechanistic framework underlying the reported antitumor effects of TCM in pancreatic cancer models.^97^^,^^114^

However, these observations must be interpreted with caution. As summarized in Table 3, the current body of evidence is derived almost exclusively from *in vitro* cell culture systems and *in vivo* animal xenograft models. Robust validation of DNA methylation–dependent effects in human pancreatic cancer tissues or large, well-controlled clinical cohorts is lacking. Future studies should prioritize distinguishing primary epigenetic targets from downstream transcriptional effects and rigorously evaluating safety, reproducibility, and clinical relevance before TCM-derived epigenetic modulators can be considered for translational application.

## 6. Targeted DNA methylation in combination with immunotherapy and molecular targeted therapy

Immunotherapy represents a transformative approach in cancer treatment by harnessing the patient’s immune system to combat tumors and establish durable antitumor immunity.^115^ However, pancreatic cancer is widely regarded as an immunologically “cold” tumor, characterized by a dense stromal barrier, limited T-cell infiltration, and a profoundly immunosuppressive tumor microenvironment. While immune checkpoint inhibitors and other immunotherapeutic agents have shown remarkable success in several malignancies, their efficacy in pancreatic cancer remains limited. Consequently, overcoming immune resistance and identifying effective combination strategies and predictive biomarkers remain major challenges.^115^ In this context, epigenetic remodeling has emerged as a promising strategy to convert immunologically “cold” tumors into “hot” ones.

### 6.1. DNA methylation and tumor immunity

Aberrant DNA methylation orchestrates immune evasion in pancreatic cancer through multiple mechanistic axes, including the suppression of antigen presentation machinery, silencing of immune-stimulatory signals, and promotion of an immunosuppressive cellular milieu.

A pivotal mechanism linking DNA methylation to antitumor immunity is the phenomenon of viral mimicry. DNA methyltransferase inhibitors (DNMTis) can induce the transcription of endogenous retroviral elements (ERVs), which are normally silenced by DNA methylation. The accumulation of ERV-derived double-stranded RNAs (dsRNAs) in the cytosol activates pattern recognition receptors such as MDA5 and RIG-I, triggering a type I interferon (IFN) response. This antiviral-like signaling cascade enhances immune cell recruitment—particularly CD8⁺ T cells and natural killer (NK) cells—into the tumor microenvironment, thereby increasing tumor immunogenicity.^28^^,^^116^

Concurrently, DNA methylation suppresses the expression of major histocompatibility complex (MHC) class I molecules and cancer-testis antigens (CTAs), impairing effective immune recognition. Demethylation restores these antigen presentation pathways, rendering tumor cells more susceptible to cytotoxic T-cell–mediated killing.^28^

In pancreatic cancer–specific contexts, emerging evidence suggests that immune-related genes are subject to aberrant methylation-dependent regulation. For example, altered DNA methylation patterns associated with PUM1 expression correlate with reduced immune cell infiltration and attenuated antitumor immune responses.^117^ Similarly, epigenetic dysregulation involving the transcription factor Twist is associated with decreased infiltration of immune effector cells, including CD8⁺ T cells and activated NK cells, thereby fostering an immunosuppressive tumor microenvironment and resistance to immune checkpoint blockade.^118^ In addition, RNA methylation modifications such as N6-methyladenosine (m6A) further modulate immune checkpoint expression and immune cell dynamics, complementing the effects of DNA methylation in shaping immunotherapy responses.^119^^,^^120^

Collectively, these findings indicate that DNA methylation constitutes a central epigenetic barrier to effective antitumor immunity in pancreatic cancer. By reversing these barriers, DNMTis can reprogram the tumor microenvironment from an immunosuppressive to an immunostimulatory state, providing a strong mechanistic rationale for combining epigenetic therapies with immunotherapy or molecular targeted treatments.

### 6.2. Combination strategies with immune checkpoint inhibitors

**Figure 3.**
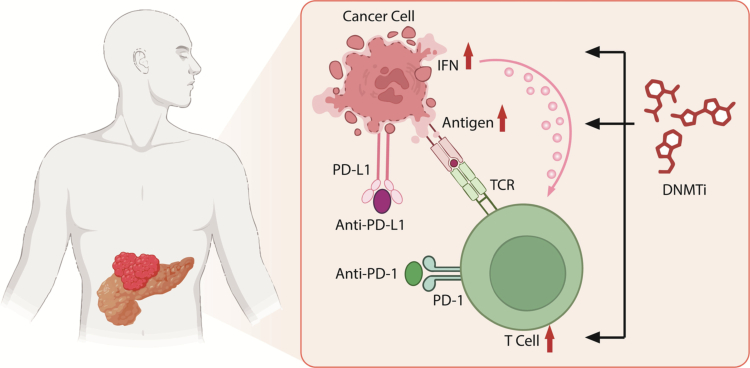
Epigenetic Priming Enhances the Efficacy of Immune Checkpoint Blockade in Pancreatic Cancer. DNA methyltransferase inhibitors (DNMTis) reprogram the immunosuppressive tumor microenvironment by inducing epigenetic remodeling in pancreatic cancer cells. DNMTi treatment promotes the expression of endogenous retroviral elements and immune-related genes, leading to activation of type I interferon (IFN) signaling and enhanced tumor antigen presentation. These changes increase tumor immunogenicity and facilitate the recruitment and activation of cytotoxic T cells. In this primed context, immune checkpoint inhibitors targeting the PD-1/PD-L1 axis exhibit improved antitumor activity through restored T cell–mediated immune recognition and effector function.

*n* recent years, combining DNA methylation–targeted therapies with immune checkpoint inhibitors (ICIs) has emerged as a promising strategy to overcome immune resistance in pancreatic cancer (Figure 3). Accumulating preclinical evidence suggests that DNA methyltransferase inhibitors (DNMTis) can function as immune sensitizers by reshaping the tumor immune microenvironment and enhancing responsiveness to ICIs.

In a seminal preclinical study, Gonda et al. demonstrated that treatment with decitabine significantly increased tumor-infiltrating T cells in pancreatic cancer mouse models. This effect was accompanied by enhanced type I interferon signaling, upregulation of antigen presentation machinery, and increased expression of immune-related genes. Importantly, decitabine synergized with PD-1/PD-L1 blockade to restore antitumor immune responses and prolong survival, whereas either treatment alone showed limited efficacy.^116^

Beyond classical nucleoside DNMTis, novel epigenetic agents targeting multiple chromatin modifiers have further expanded the therapeutic landscape. The dual DNMT1/G9a inhibitor CM272 has been shown to increase immune cell infiltration and remodel the immunosuppressive tumor microenvironment in pancreatic cancer models. When combined with anti–PD-1 therapy, CM272 produced superior antitumor effects compared with monotherapy, highlighting the advantage of simultaneously targeting DNA methylation and histone modification pathways.^87^

Although clinical evidence remains limited, early-phase clinical trials evaluating DNMTis in combination with ICIs have begun to explore the feasibility of this approach in solid tumors, including pancreatic cancer. Preliminary observations suggest that a subset of patients may derive benefit from such combinations, particularly those exhibiting epigenetically driven immune exclusion phenotypes.^28^ These findings provide a compelling rationale for further clinical investigation, emphasizing the need for biomarker-guided patient stratification and optimized dosing schedules in future trials.

### 6.3. Synergistic effects with molecular targeted therapies

Beyond immunotherapy, accumulating evidence indicates that epigenetic modulation can synergize with molecular targeted therapies by reshaping tumor signaling dependencies and DNA damage response pathways in pancreatic cancer.

KRAS mutation–driven pancreatic ductal adenocarcinoma (PDAC) exhibits distinct and dynamic DNA methylation landscapes, underscoring a close interplay between oncogenic KRAS signaling and epigenetic regulation.^50^^,^^121^ Importantly, resistance to KRAS pathway inhibitors has been associated with adaptive and reversible DNA methylation changes. Preclinical studies suggest that co-administration of DNA methyltransferase inhibitors (DNMTis) can partially reverse this epigenetically mediated resistance, thereby enhancing the antitumor efficacy of KRAS-targeted strategies.^89^

DNA methylation also critically influences therapeutic responses to DNA damage repair–targeted agents. Promoter hypermethylation of homologous recombination repair (HRR) genes, including BRCA1 and RAD51C, can induce a functional “BRCAness” phenotype analogous to that caused by germline or somatic BRCA mutations, sensitizing tumors to poly(ADP-ribose) polymerase (PARP) inhibitors.^68^ This epigenetic mechanism may potentially broaden the population of patients who could benefit from PARP inhibitor–based therapies.^122^^,^^123^ Notably, while BRCA1/RAD51C promoter methylation appears relatively infrequent in European PDAC cohorts, its higher prevalence in certain Asian populations highlights possible ethnic and molecular subtype–specific differences that warrant further investigation.^124^

Supporting this concept, preclinical studies demonstrate that combining DNMTis such as 5-azacytidine (5-AZA) with histone deacetylase (HDAC) inhibitors and PARP inhibitors synergistically enhances DNA damage accumulation, suppresses DNA repair protein expression, and induces apoptosis in pancreatic cancer cells, resulting in superior cytotoxic effects compared with monotherapy.^94^^,^^125^ Drug sensitivity analyzes further suggest that patients with low-risk tumors and relatively preserved epigenetic homeostasis exhibit improved responses to PARP inhibitors, including niraparib and olaparib.^126^

Collectively, these findings underscore the therapeutic promise of integrating DNA methylation–targeted agents with molecular targeted therapies to overcome drug resistance and improve treatment outcomes in pancreatic cancer. Future studies should prioritize the identification of predictive epigenetic biomarkers and the rational design of combination regimens to enable precision-guided therapeutic strategies.

### 6.4. RNA interference and gene editing technologies in DNA methylation regulation

RNA interference (RNAi) and gene editing technologies provide powerful experimental platforms for dissecting the causal role of DNA methylation in pancreatic cancer and offer conceptual foundations for future precision epigenetic therapies.

RNA interference (RNAi), mediated by small interfering RNAs (siRNAs) or microRNAs (miRNAs), enables sequence-specific post-transcriptional silencing of target genes through mRNA degradation or translational repression.^127^ In pancreatic cancer research, RNAi has been widely used to suppress the expression of DNA methyltransferases (DNMTs), thereby indirectly attenuating aberrant DNA methylation. For example, siRNAs targeting DNMT1 or DNMT3A/3B effectively reduce DNMT expression and enzymatic activity, leading to partial reversal of promoter hypermethylation and reactivation of tumor suppressor genes in pancreatic cancer cells.^128^^,^^129^ Beyond DNMTs, RNAi-based modulation of non-coding RNAs—including long non-coding RNAs (lncRNAs) and miRNAs—has been shown to reshape the epigenetic landscape indirectly by regulating DNMT recruitment, chromatin accessibility, or methylation-associated signaling pathways, highlighting additional layers of epigenetic control in pancreatic tumorigenesis.^128^

While RNAi primarily serves as a gene knockdown and functional validation tool, gene editing technologies—most notably the CRISPR/Cas9 system—have enabled unprecedented precision in DNA methylation manipulation. Catalytically inactive Cas9 (dCas9) fused to epigenetic modifiers, such as the DNA demethylase TET1, allows locus-specific demethylation without inducing double-strand DNA breaks. This approach facilitates direct interrogation of causal relationships between promoter methylation and gene expression.^130^

In pancreatic cancer models, CRISPR-based epigenetic editing has been employed to uncover methylation-dependent oncogenic mechanisms. Targeted demethylation of specific regulatory regions has been shown to alter tumor cell proliferation, migration, and survival, underscoring the functional importance of site-specific DNA methylation changes.^54^ Notably, CRISPR–dCas9–mediated demethylation of the ARHI promoter restored its expression, resulting in suppressed tumor growth and increased apoptosis in pancreatic cancer cells.^131^ In addition, CRISPR/Cas9 technologies have been instrumental in elucidating how oncogenic KRAS mutations reshape DNA methylation dynamics and epigenetic dependencies during pancreatic cancer progression.^50^

Although these RNAi- and CRISPR-based strategies remain largely confined to experimental and preclinical settings, they provide critical mechanistic insights and a conceptual framework for future epigenome-editing therapies. Continued advances in delivery systems, specificity, and safety will be essential for translating these technologies into clinically viable approaches for targeted epigenetic intervention in pancreatic cancer.

## 7. Current challenges and future research directions

### 7.1. Current challenges

Despite substantial progress in elucidating the role of DNA methylation in pancreatic cancer, significant challenges continue to impede clinical translation.

From a clinical perspective, the pronounced inter- and intra-tumoral heterogeneity of pancreatic cancer remains a major obstacle to the precise stratification of patients for epigenetic-targeted therapies.^50^ DNA methylation patterns vary considerably across tumor subtypes, disease stages, and individual patients, complicating biomarker standardization and therapeutic decision-making. Moreover, most existing clinical studies investigating methylation-based diagnostics or therapeutics are limited to small, single-center cohorts, lacking large-scale, multicenter validation necessary to establish robustness, reproducibility, and generalizability.^132^ In parallel, enhanced protocols for adverse event monitoring and management are required, particularly for epigenetic drugs that exert genome-wide effects, to ensure patient safety and therapeutic tolerability.^94^

Substantial challenges also persist in biospecimen acquisition and quality control. Pancreatic tumor tissue procurement is technically demanding due to anatomical constraints and often yields limited material. In addition, contamination with surrounding normal or stromal tissue can obscure tumor-specific methylation signatures, reducing analytical accuracy and interpretability.

At the technical level, current methylation detection platforms face several limitations. First, although circulating cell-free DNA (cfDNA) methylation assays show promise, their sensitivity and specificity for early-stage pancreatic cancer remain insufficient for routine clinical implementation. Second, high-throughput sequencing–based approaches, while powerful, are still associated with high costs, complex data processing, and limited accessibility in routine clinical settings. Third, reliable detection of low-abundance methylation events—particularly in early disease or minimal residual disease—remains technically challenging.

Therapeutically, currently available DNA methyltransferase inhibitors (DNMTis) exhibit broad, non-selective activity *in vivo* and lack tissue- or cell-type specificity.^133^ This global demethylation may inadvertently activate proto-oncogenes or disrupt essential regulatory programs in normal cells, leading to dose-limiting toxicities such as cytotoxicity and myelosuppression.^134^^,^^135^ These limitations have constrained the clinical efficacy of DNMTis, particularly as monotherapies in solid tumors such as pancreatic cancer.

### 7.2. Future research directions

Future research should prioritize strategies that bridge mechanistic insight with clinical applicability. First, continued optimization of DNA methylation detection technologies is essential to improve sensitivity, specificity, and reproducibility, particularly for cfDNA-based assays and early-stage disease detection. Standardization of pre-analytical workflows, assay platforms, and analytical pipelines will be critical for clinical translation.

Second, deeper mechanistic dissection of DNA methylation dynamics is needed. Integrating DNA methylation with other epigenetic layers—such as histone modifications, chromatin accessibility, RNA methylation, tumor stemness, and immune microenvironment interactions—will require single-cell and spatially resolved technologies. These approaches will enable the identification of context-dependent methylation programs and clarify how epigenetic plasticity drives therapy resistance and immune evasion.

Third, rigorous validation of methylation-based biomarkers and therapeutic strategies through large-scale, multicenter clinical trials is indispensable. Such efforts should incorporate diverse patient populations to account for ethnic, genetic, and tumor subtype–specific variability, thereby enhancing translational relevance.

Multi-omics integration—including genomics, epigenomics, transcriptomics, proteomics, and metabolomics—offers unprecedented opportunities to construct comprehensive molecular maps of pancreatic cancer progression and treatment response.^136^^,^^137^ In this context, artificial intelligence (AI) and machine learning approaches are poised to play a transformative role by enabling clinically actionable integration of complex, high-dimensional datasets.^138^ When combined with advanced imaging, genomic profiling, and real-world clinical data, AI-driven frameworks may substantially improve diagnostic precision, patient stratification, and personalized therapeutic design.^139^

Ultimately, interdisciplinary collaboration across molecular biology, clinical oncology, bioinformatics, and data science will be essential to maximize research efficiency and translational impact. The integration of DNA methylation research with AI-powered analytics and big data infrastructures is expected to drive major advances in precision oncology, paving the way for more effective diagnosis, treatment, and prevention strategies in pancreatic cancer.^139^^,^^140^

## 8. Conclusion

Accumulating evidence over the past decade has established dysregulated DNA methylation as a central and dynamic driver of pancreatic cancer initiation, progression, and therapeutic resistance. Advances in high-throughput sequencing and methylome profiling have enabled the systematic identification of methylation-based biomarkers with potential utility in early diagnosis, molecular subtyping, prognostic stratification, and prediction of treatment response. The emergence of liquid biopsy approaches, particularly cfDNA methylation analysis, has further expanded opportunities for non-invasive disease detection and longitudinal monitoring.

Despite these advances, the clinical translation of DNA methylation–based strategies remains constrained by tumor heterogeneity, limited therapeutic specificity, and the risk of off-target effects associated with global epigenetic modulation. These challenges underscore the necessity of moving beyond one-size-fits-all approaches toward precision epigenetic interventions guided by robust biomarkers and mechanistic insight.

Importantly, DNA methylation is increasingly recognized not merely as a passive epigenetic marker, but as an actionable and reversible regulatory layer that intersects with oncogenic signaling, tumor stemness, DNA damage repair, and immune evasion. This unique position makes methylation an attractive therapeutic nexus. Rational combination strategies—integrating DNMT inhibitors with immunotherapy, molecular targeted agents, or epigenome-editing technologies—offer promising avenues to overcome resistance and reshape the pancreatic tumor microenvironment.

Looking ahead, the convergence of single-cell and spatial epigenomics, multi-omics integration, and artificial intelligence–driven analytics is expected to accelerate the identification of clinically actionable methylation signatures and optimize patient stratification. Continued interdisciplinary collaboration among basic scientists, clinicians, and data scientists will be essential to translate these discoveries into safe, effective, and personalized therapeutic strategies.

In summary, aberrant DNA methylation represents both a fundamental biological hallmark and a translational opportunity in pancreatic cancer. Harnessing its diagnostic, prognostic, and therapeutic potential holds promise for improving early detection, refining treatment selection, and ultimately enhancing clinical outcomes for patients with this highly lethal disease.

## Data Availability

Data sharing is not applicable to this article as no data sets were generated or analyzed during the current study.
